# Validation of *in vitro* methods for human cytochrome P450 enzyme induction: Outcome of a multi-laboratory study

**DOI:** 10.1016/j.tiv.2019.05.019

**Published:** 2019-10

**Authors:** Camilla Bernasconi, Olavi Pelkonen, Tommy B. Andersson, Judy Strickland, Iwona Wilk-Zasadna, David Asturiol, Thomas Cole, Roman Liska, Andrew Worth, Ursula Müller-Vieira, Lysiane Richert, Christophe Chesne, Sandra Coecke

**Affiliations:** a European Commission, Joint Research Centre (JRC), Ispra, Italy; b Research Unit of Biomedicine/Pharmacology and Toxicology, Faculty of Medicine, Aapistie 5B, University of Oulu, FIN-90014, Finland; c Drug Metabolism and Pharmacokinetics, Cardiovascular, Renal and Metabolism, IMED Biotech Unit, AstraZeneca, Gothenburg, Sweden; d Department of Physiology and Pharmacology, Section of Pharmacogenetics, Karolinska Institutet, SE-171 77 Stockholm, Sweden; e Integrated Laboratory Systems (contractor supporting NICEATM), Research Triangle Park, North, Carolina, 27709, USA; f Boehringer Ingelheim, Germany. Department of Drug Discovery Sciences, Boehringer Ingelheim Pharma GmbH & Co. KG, Biberach, an der Riss, Germany; g KaLy-Cell, 20A, rue du Général Leclerc, 67115 Plobsheim, France^g^ Biopredic International, Parc d’activité de la Bretèche Bâtiment A4, 35760 Saint Grégoire, France; h Biopredic International, Parc d’activité de la Bretèche Bâtiment A4, 35760 Saint Grégoire, France; i Clinical Research Center, Oulu University Hospital, Finland

**Keywords:** CYP induction, human hepatocytes, HepaRG, metabolism, *in vitro* hepatic system, validation

## Abstract

CYP enzyme induction is a sensitive biomarker for phenotypic metabolic competence of *in vitro* test systems; it is a key event associated with thyroid disruption, and a biomarker for toxicologically relevant nuclear receptor-mediated pathways. This paper summarises the results of a multi-laboratory validation study of two *in vitro* methods that assess the potential of chemicals to induce cytochrome P450 (CYP) enzyme activity, in particular CYP1A2, CYP2B6, and CYP3A4. The methods are based on the use of cryopreserved primary human hepatocytes (PHH) and human HepaRG cells.

The validation study was coordinated by the European Union Reference Laboratory for Alternatives to Animal Testing of the European Commission's Joint Research Centre and involved a ring trial among six laboratories. The reproducibility was assessed within and between laboratories using a validation set of 13 selected chemicals (known human inducers and non-inducers) tested under blind conditions. The ability of the two methods to predict human CYP induction potential was assessed. Chemical space analysis confirmed that the selected chemicals are broadly representative of a diverse range of chemicals.

The two methods were found to be reliable and relevant *in vitro* tools for the assessment of human CYP induction, with the HepaRG method being better suited for routine testing. Recommendations for the practical application of the two methods are proposed.

## Highlights

• Paper summarises the results of a multi-laboratory validation study.
• Two human derived metabolically competent test systems used.
• *In vitro* methods validated as reliable and relevant for CYP enzyme induction assessment.
• Two *in vitro* methods can support regulatory chemical risk assessment with HepaRG-based method better suited for routine chemical testing.
• CYP enzyme induction biomarker for thyroid and nuclear receptor-mediated disruption.

## 1 Introduction

The toxicity profile of an exogenous chemical (xenobiotic) to which the body is exposed depends not only on the toxicity of the parent compound, but also on any toxicologically relevant metabolites that may be formed during metabolism and on the xenobiotic's ability to induce biotransformation enzymes that affect its rate of metabolism (Tsaioun et al., 2016). Information on metabolism, including metabolic activation by CYP induction, is useful in toxicity testing strategies, for example to support *in vitro* to *in vivo* extrapolation (Coecke et al., 2005; Coecke et al., 2006; Wilk-Zasadna et al., 2015).

Over the last two decades, considerable progress has been made in developing *in vitro* metabolism methods based on human test systems (Coecke et al., 2013; Donato et al., 2008; Vermeir et al., 2005; Vinken and Hengstler, 2018). However, there were no formally validated test systems based on intact functional human hepatic cells capable of maintaining key metabolic activity functions for up to 3 days in culture.

Of all xenobiotic-metabolising enzymes, the Cytochrome (CYP) P450 enzymes are of particular importance due to their abundance and functional versatility (Raunio et al., 2015). They may transform a xenobiotic into a harmless metabolite (detoxification) or, *vice versa,* a non-toxic parent compound into a toxic metabolite. Besides detoxifying xenobiotics, CYP enzymes play a key role in the biosynthesis of endogenous substrates such as steroid hormones, prostaglandins and bile acids. Therefore, xenobiotic CYP enzyme induction may cause dysregulation of normal metabolism and homeostasis, with potential toxicological effects (Staudinger et al., 2013; Amacher, 2010).

CYP enzyme induction has been selected as the biological endpoint to validate cryopreserved primary human hepatocytes and the cryopreserved human HepaRG cell line (hereafter referred to as PHH and HepaRG cells, respectively) as reliable hepatic metabolically competent test systems, as it requires the whole molecular machinery (i.e. receptor and transporter expression, transcription, translation and expression of functional CYP enzymes) to be present and functional in the test system.

At the molecular level, CYP enzyme induction is a rather slow process, controlled by a set of nuclear receptors associated with downstream signal transduction pathways. The process is initiated by the binding of endogenous or exogenous ligand(s) to specific nuclear receptors/transcription factors^1^, namely the aryl hydrocarbon receptor (AhR), the constitutive androstane receptor (CAR), and the pregnane X receptor (PXR). AhR, PXR and CAR are primarily responsible for inducing transcription of the CYP1A, CYP3A and CYP2B families, respectively (Hakkola et al., 2018). In addition to mediating detoxification, CAR, PXR and AhR have been implicated in the regulation of a broader range of physiological functions (Kretschmer and Baldwin, 2005; Sueyoshi et al., 2014; Wang and Tompkins, 2008), where dysregulation can lead to adverse effects (Hakkola et al., 2018) such as inflammation (Rubin et al., 2015; Christmas, 2015), cholestasis, steatosis (Gómez-Lechón et al., 2009), hepatotoxicity (Woolbright and Jaeschke, 2015), carcinogenesis (De Mattia et al., 2016; Pondugula et al., 2016; Fucic et al., 2017), and thyroid disruption (Patrick, 2009). Thus, in these cases, CYP induction serves as a biomarker for key events associated with adverse health effects.

In the area of regulatory toxicology, validation plays an indispensable role by independently establishing the relevance and reliability of a method for a specific purpose (Hartung et al., 2004), thereby promoting its regulatory acceptance. With a view to promoting the uptake of CYP induction methods in the regulatory assessment of chemicals, the European Union Reference Laboratory for Alternatives to Animal Testing (EURL ECVAM, 2014) of the European Commission's Joint Research Centre (JRC) organised a multi-laboratory validation study to assess the ability of two human *in vitro* metabolically competent test systems, namely PHH and HepaRG cells, to reliably predict the induction status of CYP1A2, CYP2B6 and CYP3A4 upon exposure to selected chemicals (i.e. known human *in vivo* inducers and non-inducers, see Table 1). The selected CYP enzymes are globally accepted as alternative biomarkers of CYP induction in the regulatory guidelines of pharmaceutical agencies (FDA, 2017; EMA, 2012). They are also expressed in human liver and human intestine (Lindell et al., 2003) and are inducible by well-established reference chemicals (Lehmann et al., 1998; Gibson et al., 2002; Chen et al., 2004; Wang et al., 2004; Sueyoshi et al., 1999;

**Table 1:** Test items.

#	Chemical Name	CAS#	MW	CYP induction *in vivo* literature reference^1^	CYP induction *in vitro* (human hepatocytes)^2^
1	Omeprazole	73590-58-6	345.4	**CYP 1A2** NOT induced at clinically used doses (Rost et al., 1994; Rost et al., 1999; Sarich et al., 1997; Andersson et al., 2001) **CYP2B6** NO studies **CYP3A4** NOT induced (Rost et al., 1994; Andersson et al., 2001; Zhou et al., 2018)	CYP1A2, CYP2B6, CYP3A4
2	Carbamazepine	298-46-4	236.3	**CYP 1A2** (Parker et al., 1998; Lucas et al., 1998; Oscarson et al., 2006) **CYP2B6** (Ketter et al., 1995; Ji et al., 2008) **CYP 3A4** (Moreland et al., 1982; Crawford et al., 1990; Herman et al., 2006; Perucca et al., 2004)	CYP1A2, CYP2B6, CYP3A4
3	Phenytoin sodium	630-93-3	274.3	**CYP1A2**Wietholtz et al., 1989; Miller et al., 1990) **CYP2B6** (Slattery et al., 1996; Williams et al., 1999) **CYP3A4** (Werk Jr et al., 1964; Crawford et al., 1990)	CYP1A2, CYP2B6, CYP3A4
4	Penicillin G sodium	69-57-8	356.4	NO evidence of induction found; unlikely because of PK characteristics	No
5	Indole-3-carbinol	700-06-1	147.2	**CYP 1A2** (Reed et al., 2005) **CYP2B6/CYP3A4** NO studies	CYP1A2
6	Rifabutin	72559-06-9	847.0	**CYP1A2** NOT induced (Gillum et al., 1996) **CYP2B6** NO studies **CYP3A4 (**Horita and Doi, 2014; Barditch-Crovo et al., 1999)	CYP1A2, CYP3A4
7	Sulfinpyrazone	57-96-5	404.5	**CYP1A2** (Birkett et al., 1983) **CYP2B6** NO studies **CYP3A4** (Pichard et al., 1990; Birkett et al., 1983; Wing et al., 1985; Walter et al., 1982; Staiger et al., 1983)	CYP1A2, CYP2B6, CYP3A4
8	Bosentan hydrate	157212-55-0	569.6	**CYP1A2** NO studies **CYP2B6** NO studies **CYP 3A4** (Weber et al., 1999a, Weber et al., 1999b; van Giersbergen et al., 2002a, van Giersbergen et al., 2002b; Dingemanse and van Giersbergen, 2004)	CYP3A4
9	Artemisinin	63968-64-9	282.3	**CYP1A2** NO studies **CYP 2B6** (Simonsson et al., 2003; Elsherbiny et al., 2008) **CYP 3A4** (Asimus et al., 2007) No induction (Svensson et al., 1998)	CYP1A2, CYP2B6, CYP3A4
10	Efavirenz	154598-52-4	315.7	**CYP1A2** NO studies **CYP2B6** (Robertson et al., 2008; Ngaimisi et al., 2010) **CYP3A4** (Mouly et al., 2002; Fellay et al., 2005)	CYP2B6, CYP3A4
11	Rifampicin	13292-46-1	822.9	**CYP1A2** (Robson et al., 1984; Wietholtz et al., 1995; Backman et al., 2006) **CYP2B6** (López-Cortés et al., 2002; Loboz et al., 2006) **CYP3A4** (Ohnhaus and Park, 1979; Barditch-Crovo et al., 1999; Lin, 2006; Kanebratt et al., 2008)	CYP1A2, CYP2B6, CYP3A4
12	Metoprolol	51384-51-1	267.4	NO evidence of induction in clinical use, but a small fraction is metabolized in vitro by CYP3A4, CYP2B6 and CYP2C9 (Berger et al., 2018).	No
13	Sotalol hydrochloride	959-24-0	308.8	NO evidence of induction found; unlikely because of PK characteristics (Yamreudeewong et al., 2003)	No

(1) Further information and references from Washington University database https://www.druginteractioninfo.org/); also Hoffmann et al. (2014), Pelkonen et al. (2008) and Hukkanen et al. (2012).

(2) Individual references in Abadie et al. (2009), Richert et al. (2010), Preissner et al. (2010) (Database address: http://bioinformatics.charite.de/supercyp) and in Hoffmann et al. (2014).

).

This paper describes the organisation and execution of the EURL ECVAM validation study and presents the results obtained. The validation study was conducted in accordance with several reliability considerations (e.g. serum free medium, inclusion of reference items) detailed in the recently published OECD guidance document on good *in vitro* method practices (GIVIMP) (OECD, 2018).

## 2 Materials and methods

### 2.1 Choice of test systems for *in vitro* metabolism methods

The *in vitro* test systems available for human metabolism studies include human intact cells (tissue slices, isolated and cultured hepatocytes, liver cell lines) and subcellular fractions (microsomes, recombinant enzymes) (Coecke et al., 2006; Donato et al., 2008; Vermeir et al., 2005). Among these possible test systems, PHH and HepaRG cells were identified as the most promising to include in the validation study (Mandenius et al., 2011; Vitrocellomics project https://cordis.europa.eu/project/rcn/85218/reporting/en). PHH contain all drug/xenobiotic metabolising enzymes and cofactors, and are considered relevant for a variety of toxicokinetic and toxicodynamic applications where it is important to account for inter-individual variation (Godoy et al., 2013). Similarly, the HepaRG cell line maintains metabolic capacity comparable to human hepatocytes, including expression of liver metabolising enzymes, nuclear receptors, and hepatic xenobiotic transporters (Aninat et al., 2006; Le Vee et al., 2006; Turpeinen et al., 2009; Andersson et al., 2012).

In addition to biological relevance, practical considerations were also important in the choice of test systems. Recent developments in cell cryopreservation and optimisation of seeding conditions have facilitated continuity of commercial supply for both PHH and HepaRG cells. The responses of both cryopreserved PHH and HepaRG to specific inducers are similar to those of freshly isolated PHH (Abadie-Viollon et al., 2010; Aninat et al., 2006; Alexandre et al., 2012; Andersson et al., 2012). Availability of chemically defined culture media allows these test systems to be maintained without the use of foetal calf serum thereby avoiding undefined media components and increasing the reproducibility of culture conditions (OECD, 2018).

### 2.2 Organisation of the validation study

**Fig. 1:**
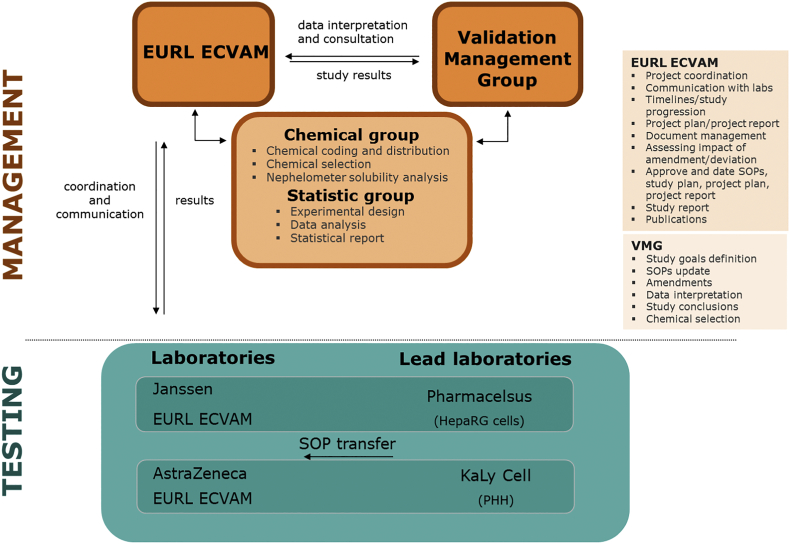
Validation study organisation. Pharmacelsus (Lab 1) Janssen (Lab 2) and ECVAM (Lab 3) for HepaRG cells; Kaly Cell (Lab 4) AstraZeneca (Lab 5) and ECVAM (Lab 6) for PHH.

The validation study was conducted as a ring trial among six laboratories. Four were already technically proficient in one of the methods (KaLy-Cell and AstraZeneca for PHH; Pharmacelsus and Janssen Pharmaceutica for HepaRG cells). In addition, EURL ECVAM participated for both methods, effectively acting as two laboratories (Fig. 1). Independent experts with a supervisory role formed a validation management group (VMG), with international organisations for validation of alternative methods were also represented [NICEATM/ICCVAM (USA) and JaCVAM (Japan)]. Management of the set of validation chemicals (i.e. acquisition, coding and distribution) and the statistical data analysis were carried out by EURL ECVAM.

Following the modular principles of validation (Hartung et al., 2004; OECD, 2005), the scope of the study included test definition, within-laboratory reproducibility (WLR), transferability, and between laboratory reproducibility (BLR). The methods were first evaluated by the lead laboratories (KaLy-Cell for PHH, and Pharmacelsus for HepaRG cells) to confirm standard operating procedures (SOPs). The subsequent training and transfer to the other participating laboratories was then followed by testing a validation set of 13 selected chemicals by all six laboratories for BLR. For both PHH and HepaRG cells, three biological replicates (different cell batches) were run by each laboratory.

### 2.3 Chemical selection

To provide insight into the predictive capacities of the evaluated *in vitro* methods, a principal requirement for chemical selection was the availability of adequate (relevant and reliable) human *in vivo* data sets. Key data sources used were comprehensive review articles (Pelkonen et al., 2008; Hukkanen, 2012) and a database hosted by Washington University (https://www.druginteractioninfo.org/). Original references cited in the review articles were also compiled for the record.

An important practical consideration was that there was only a limited set of pharmaceuticals with adequate *in vivo* human reference data for each of the CYP isoforms investigated. This is the reason why the chemical selection was limited to pharmaceuticals with *in vivo* human data available from clinical monitoring. For the purpose of the CYP induction validation study, a validation set of 13 selected chemicals (Table 1) was used to assess the predictive capacity of the two *in vitro* methods.

Furthermore, specific reference items with known induction potential for CYP1A2, CYP2B6, and CYP3A4 are indicated in Table 2. These reference items are used to calculate the response to the blind coded validation set of chemicals. For both CYP induction methods acceptance criteria for the reference items (acting also as positive control items) were established to provide evidence that the PHH and the HepaRG cells are responsive under the actual condition of the two *in vitro* methods (OECD, 2004, 2018; https://tsar.jrc.ec.europa.eu/test-method/tm2009-13 and https://tsar.jrc.ec.europa.eu/test-method/tm2009-14).

**Table 2:** Reference items, with the respective CYP induction isoform and relevant exposure concentration.

CYP	Reference item for human CYP induction	Enzymatic probe substrate	Concentration in the cocktail (μM)	Metabolite measured
1A2	β-naphthoflavone (BNF) 25 μM	Phenacetin	10 (PHH)-26 (HepaRG)	Acetaminophen
2B6	Phenobarbital (PB) 500 μM	Bupropion	100	OH-bupropion
3A4	Rifampicin (RIF) 10 μM	Midazolam	3	1-OH-midazolam

### 2.4 Chemical space analysis

The chemical space covered by the validation set of 13 chemicals and the 3 reference items (with rifampicin being both a blind coded validation chemical and a reference item) is represented by showing the position of these in a similarity space formed by other chemicals found in relevant lists (e.g. REACH registered substances downloaded from ECHA (2017), approved drugs from the *Drugbank database* (2018), and Tox21 chemicals NIH (2018)).

To determine chemical similarity, Tanimoto similarity analysis (Bajusz et al., 2015) was applied. The Tanimoto similarity metric between two chemicals is based on the number of 2D structural features they have in common compared with the total count of structural features that are used by the selected fingerprints that represent the molecules.

The chemical space analysis was performed post-hoc and did not affect the CYP induction validation data analysis or the *in vitro* classification of validation set of chemicals.

### 2.5 The human CYP enzyme induction *in vitro* method

The Standard Operating Procedures (SOPs) for the two methods are available from the EURL ECVAM Tracking System for Alternative methods towards Regulatory acceptance (TSAR) where they are indicated as *in vitro* method No. 193 (https://tsar.jrc.ec.europa.eu/test-method/tm2009-13 for PHH) and No. 194 (https://tsar.jrc.ec.europa.eu/test-method/tm2009-14 for HepaRG cells). TSAR provides an overview of alternative (non-animal) methods that have been proposed for regulatory safety or efficacy testing.

The validation set of 13 selected chemicals and reference item stock solutions were prepared in dimethyl sulfoxide (DMSO) and further diluted in the appropriate culture medium^2^ to achieve a final DMSO concentration of 0.1% (v/v). Experimental negative control culture wells (in triplicate) were treated with solvent only [DMSO 0.1% (v/v)].

For each chemical of the validation set, only soluble and non-cytotoxic concentrations were tested in triplicate in the CYP enzyme induction experiments. Therefore, before assessing CYP enzyme induction potential, solubility and cytotoxicity were separately and independently assessed by the six laboratories (with EURL ECVAM representing two test facilities for HepaRG cells and PHH).

#### 2.5.1 Solubility

The SOPs prescribed 40 mg/mL as the initial concentration for determination of solubility. The SOPs relied on visual inspection for solubility observation. In case of apparent insolubility in DMSO or precipitation in medium, dissolution was attempted by incremental two-fold dilution (20, 10, 5 mg/ml) as necessary. The absence of precipitation in the medium was checked pre- and post-incubation (24 hours) by centrifugation of the sample and observation of any precipitation (pellet residue).

In addition to the visual inspection performed by the validation laboratories as described in the SOP, EURL ECVAM introduced nephelometry for systematic solubility determination of the 13 validation set chemicals. Nephelometry uses a laser beam and the principle of Tyndall effect light scatter to detect turbidity due to insolubility. The nephelometer method used formazin as reference item (1, 5, 10 and 20 nephelometric turbidity units (NTU)) to calculate the relative turbidity (RTU) of the validation set of chemicals. To the naked eye, 20 NTU is perceptible, while the nephelometer was sensitive to 5 NTU, with 1 NTU equivalent to background (solvent/medium blank).

A definition for solubility was adopted by setting 5 and 10 NTU formazin reference items as turbidity thresholds. Effectively, for stock solutions and medium dilutions, relative turbidity equivalent to <10 NTU was defined 'soluble' while >10 NTU was defined as 'insoluble'. Considering instrument sensitivity, turbidity between 5 and 10 NTU was refined as 'solubility limit' (still effectively 'soluble').

However, since the nephelometry was an extension to the project, with definitive results only available at a later stage, solubility of the validation set of chemicals for the *in vitro* experiments was concluded only from the visual inspections done by the validation laboratories.

#### 2.5.2 Cytotoxicity

Potential cytotoxicity of the validation set chemicals for PHH and HepaRG cells was determined starting from the highest soluble concentration, followed by a 1:1 or 1:3 dilution for PHH and HepaRG cells, respectively. The incubation time reflected the conditions used for the induction assays (72 hours and 48 hours of incubation for PHH and HepaRG cells, respectively). The cytotoxicity assay was based on the conversion of redox dye resazurin to fluorescent resorufin by living cells. Non-viable cells, without metabolic capacity, yield no fluorimetry signal.

Results were expressed as fractional survival (FS %) with respect to untreated controls and were calculated based on measured relative fluorescent units (RFU), corrected for the background signal:

$$\%\mathrm{FS}=\frac{{\mathrm{RFU}}_{\mathrm{treated cells}}-\mathrm{mean}\;{\mathrm{RFU}}_{\mathrm{background}}}{{\mathrm{RFU}}_{\mathrm{untreated cells}}-\mathrm{mean}\;{\mathrm{RFU}}_{\mathrm{background}}}\;x\;100$$

Cytotoxicity was evaluated from the dose-response curve, where the mean FS (%) of three technical replicates was plotted versus the corresponding concentration.

The SOP acceptance criteria required a cell viability of 50-70 % for doxorubicin (8 μM, HepaRG cells) and ≤70 % for chlorpromazine (25 μM, PHH), the cytotoxicity reference items. The highest concentration with viability reduction <20% (PHH) or <10% (HepaRG) was eligible as starting concentration for the induction assay.

#### 2.5.3 CYP enzyme induction assay

The CYP induction assays involved exposure to the validation set of chemicals at 6 serial dilutions (1:3 ratio) over 72 hours (PHH) or 48 hours (HepaRG cells) with medium renewal every 24 hours. The HepaRG cells and PHH assays included three technical replicates (triplicate of validation set chemicals) repeated with three biological replicates (different cell batches or donors). Parallel assay of the reference items at appropriate concentrations (Table 2) provided experimental positive controls. Cells exposed to solvent (i.e. 0.1 % DMSO) diluted in medium served as the negative control.

**Fig. 2:**
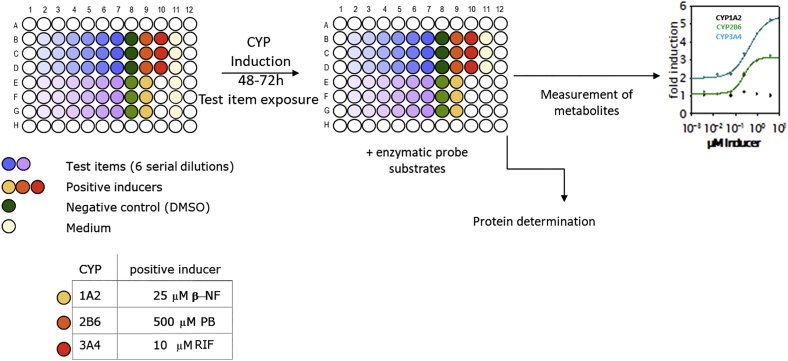
CYP induction experimental design.

CYP enzyme activity was determined by applying fresh medium containing a combination (“cocktail”) of the CYP-selective probe substrates phenacetin (CYP1A2), bupropion (CYP2B6) and midazolam (CYP3A4)^3^ (Fig. 2). Plate formats (48-well for PHH and 96-well for HepaRG cells) and exposure times (72h for PHH and 48h for HepaRG cells) were previously optimised for sensitivity of the test systems to potential inducers. The plate layouts allowed triplicate testing of two validation set chemicals at six concentrations.

For quantitative analysis of CYP enzyme activity, the formation of specific products by the respective isoenzyme (Table 2) namely acetaminophen (CYP1A2), hydroxybupropion (CYP2B6) and 1-hydroxymidazolam (CYP3A4) was quantified by liquid chromatography–mass spectrometry (LC/MS) analysis. Different LC/MS systems (e.g. Varian, Thermofisher, Waters) were used by the participating laboratories. Prior to routine operation, the different LC/MS instruments in use by the laboratories were to be confirmed as compliant with required the performance criteria. LC/MS analytical method protocols for metabolite quantification were validated for accuracy, precision, lower and upper limits of quantitation (LLOQ and ULOQ, respectively) and method linearity, consistent with guidelines of the European Medicines Agency (EMA, 2011), the updated guidelines of the Food and Drug Administration (FDA, 2018) and the OECD (OECD, 2018). LLOQ of 2.30 nM for acetaminophen, 1.15 nM for hydroxybupropion and 1.15 nM for 1-hydroxymidazolam were required before proceeding with sample analysis. Griseofulvin or 5.5-diethyl-1.3-diphenyl-2-iminobarbituric acid were included as reference items allowing correction for any loss of analyte during sample preparation and sample injection.

Analytical assay acceptance criteria were adapted from FDA Guidance for Industry Bioanalytical Method Validation (2001) and from Shah et al. (2000) and Viswanathan et al. (2007).

Quantitative analytical data for the specific products were normalised per protein content per well. Protein quantification was assessed by the Pierce Bicinchoninic Acid method. The protein concentration of the tested sample was interpolated from a bovine serum albumin standard curve in 0.1 M NaOH using the linear regression where the standard curve is a plot of the average blank-corrected absorbance for each standard vs. its concentration in mg/ml. Interpolated data are accepted as long as the coefficient of determination (R^2^) for the linear regression is equal or greater than 0.9, in accordance with the SOP.

### 2.6 Data analysis

The inclusion of relevant reference and control items, and setting of acceptance criteria for performance on the basis of historical data, is essential for regulatory applicability of *in vitro* methods (OECD, 2018). The acceptance criteria used in the validation study are explained in detail in the specific method SOPs (https://tsar.jrc.ec.europa.eu/test-method/tm2009-13 and https://tsar.jrc.ec.europa.eu/test-method/tm2009-14).

#### 2.6.1 Evaluation of CYP enzyme induction

Each induction plate included wells for the measurements of basal CYP1A2, 2B6 and 3A4 activities (i.e. cells exposed to the negative control (0.1% DMSO)) and of reference items induced activities. The CYP enzyme induction activity results were expressed as CYP activities in pmol of specific products/min/mg protein.

The induction potential of the validation set chemicals and the reference items was calculated as n-fold increase relative to the negative control (0.1% DMSO) averaged over the three replicates:

$n‐\text{fold}\;\mathrm{CYP}\;\text{induction}=\frac{\text{Validation}\;\mathrm{set}\;\text{chemical}\;\mathrm{CYP}\;\text{activity or Reference item}\;\mathrm{CYP}\;\text{activity}}{\text{Negative control}\;\mathrm{CYP}\;\text{activity}}$

One key acceptance criterion for each assay was that reference items (positive controls) were required to produce ≥ 2-fold CYP induction with respect to enzyme basal activity. A validation set chemical with ≥2-fold induction potential has been described as an *in vitro* positive inducer (Kanebratt and Andersson, 2008). However, based on the validation study results, to ensure consistency (avoiding false positives), it was also required to have at least two consecutive concentrations in the dose-response generating ≥ 2-fold induction to classify test items as *in vitro* positive inducers.

#### 2.6.2 Reproducibility

The capability of an *in vitro* method to provide reliable results is an important characteristic evaluated in validation studies. For the CYP enzyme induction method the focus was mainly on comparison of assigned classifications across different batches (between-batch reproducibility; BBR) and across laboratories (between-laboratory reproducibility; BLR).

For a given CYP, two reproducibility measures based on assigned classifications were evaluated. First, the reproducibility of results across three batches (BBR) was evaluated for each laboratory. Secondly, the reproducibility of results across three participating laboratories (BLR) for a given batch was assessed.

More precisely, we define BBR_L_ and BLR_B_ measures as follows:

• BBR_L_ represents the percentage of validation set chemicals that have concordant classifications across three batches tested in laboratory L.
• BLR_B_ represents the percentage of validation set chemicals that have concordant classifications across three participating laboratories for batch B.

In addition to the measures above, the aggregated measure BBR and BLR is constructed as an average across three laboratories and batches, respectively.

The BBR was used a proxy for within-laboratory reproducibility (WLR) which could not be directly evaluated in certain cases. Particularly in the case of PHH, batches were provided only once. However, this is not considered to be a shortcoming, since the BLR can be regarded as the more conservative (lower) estimate of reproducibility.

#### 2.6.3 Relevance (predictive capacity)

Comparison of the study results to human CYP induction used a ratio of *in vivo* plasma concentrations (Cmax) to *in vitro* concentrations producing 2-fold induction (F2 values) (Weiss and Haefeli, 2006; Kanebratt and Andersson, 2008; Grime et al., 2010). A ratio of >0.5 was the criterion used to predict an *in vivo* CYP enzyme induction response. This is a rather conservative threshold, implying that an *in vitro* concentration resulting in 2-fold induction was significant at half the Cmax value. Alternatively, Cmax/EC_50_ values could be used, but for some of the 13 validation set chemicals a full dose-response curve for calculation of an EC_50_ was not attained. The human *in vivo* classifications (inducer, non-inducer) for the validation set chemicals were based on literature data (Table 1).

To classify each chemical as a positive *in vitro* inducer, a positive induction result in one donor (PHH) or batch (HepaRG cells) in each of the three laboratories was required. This criterion is just a very cautious interpretation of FDA guidance (FDA, 2017).

## 3 Results

### 3.1 Solubility and cytotoxicity

#### 3.1.1 Solubility

The six laboratories uniformly reported 40 mg/ml in DMSO stock solution as soluble for 12 of the 13 validation set chemicals. The exception, phenytoin, was soluble at 40 mg/ml using a 1:1 blend of DMSO with water. The stock solution observations were confirmed by nephelometry, where only background signals equivalent to solvent blank were measured.

**Fig. 3:**
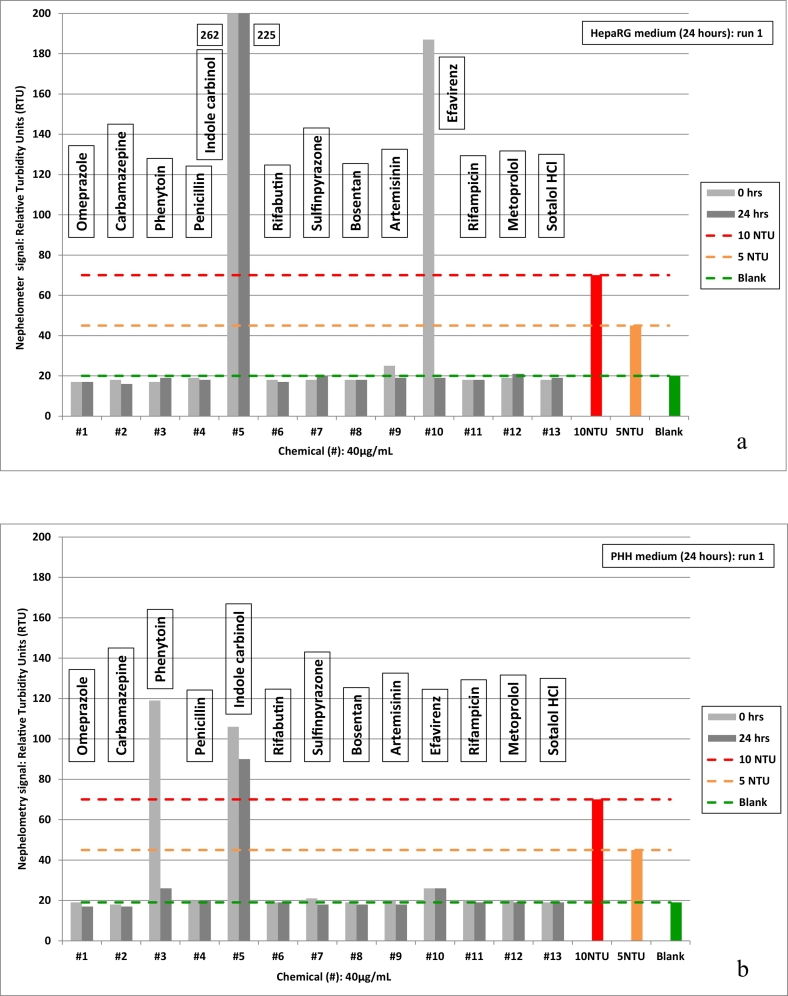
Relative turbidity (RTU) of test item chemicals at 40 μg/mL in assay medium compared to formazin reference standards (5 and 10 NTU) by nephelometry. a) HepaRG medium; b) PHH medium.

For the dilutions in medium, 40 μg/ml was consistently observed among the laboratories to be stable for 8 of the validation set chemicals, while some discordance of solubility was reported for the others (Table 3). In particular, nephelometry detected insoluble suspensions for indole carbinol, efavirenz and phenytoin, illustrated by turbidity graphs for the two media (Fig. 3).

**Table 3:** Test items (5 of 13) with differences in solubility by visual observation (Labs) vs. nephelometry (ECVAM) with DMSO stock solution (regular type) vs. assay medium (bold type) ^1^ DMSO+water (1:1) blend; ^2^ solubility limit.

**#**	Chemical Name	Lab 1	Lab 2	Lab 3	ECVAM	Lab 4	Lab 5	Lab 6	ECVAM
		DMSO stock solution solubility (mg/mL)							
		HepaRG medium stability (μg/mL)				PHH medium stability (μg/mL)			
3	Phenytoin sodium	40 ^1^	20	40	40 ^1^	40	20	10	40 ^1^
		**40**	**20**	**40**	**40**	**40**	**20**	**10**	**20**
4	Penicillin G sodium	40	40	40	40	40	40	40	40
		**40**	**40**	**40**	**40**	**40**	**40**	**20**	**40**
5	Indole carbinol	40	40	40	40	40	40	40	40
		**5**	**10**	**10**	**10**^**2**^	**10**	**20**	**20**	**10**^**2**^
6	Rifabutin	40	40	40	40	40	40	40	40
		**40**	**20**	**40**	**40**	**40**	**40**	**40**	**40**
10	Efavirenz	40	40	40	40	40	40	40	40
		**20**	**40**	**40**	**20**	**40**	**40**	**40**	**40**^**2**^

Precipitation of indole carbinol, notably intense in HepaRG medium at 40 μg/ml was also persistent at 20 μg/ml, and even perceptible at 10 μg/ml in both media based on nephelometer measurements. Relative turbidity at 10 μg/ml was 5 NTU < RTU value < 10 NTU equivalents.

At 40 μg/ml significant precipitation was also observed for efavirenz in HepaRG medium and phenytoin in PHH medium, although only initially (time zero). Repeat measurements indicated borderline solubility limit for efavirenz at 40 μg/ml in PHH (post-incubation). Also in PHH, trace turbidity was evident for phenytoin at 20 and 10 μg/ml (pre-incubation). Based on the nephelometry measurements, efavirenz was concluded soluble at 20 μg/ml in HepaRG medium with a solubility limit at 40 μg/ml in PHH medium. Conversely, phenytoin was concluded soluble in HepaRG medium at 40 μg/ml and in PHH medium at 20 μg/ml.

Based on the visual inspection observations available at the time of the *in vitro* method implementation, the VMG excluded indole carbinol from the testing program due to uncertain solubility (Table 3).

#### 3.1.2 Cytotoxicity

The maximum soluble and non-cytotoxic concentrations applicable to the CYP induction *in vitro* methods for all 13 validation set chemicals are shown in Table 4. For HepaRG cells, rifabutin and efavirenz were cytotoxic based on the SOP acceptance criteria and therefore excluded from the CYP enzyme induction assay. For PHH, rifabutin, bosentan and efavirenz were tested for *in vitro* CYP induction at starting concentrations of 20, 10, and 2.5 μg/ml, respectively.

**Table 4:** Summary of the highest soluble non cytotoxic concentrations used for the subsequent induction assay. Solvent used for phenytoin sodium was a 1:1 blend DMSO:water. All the other test items were dissolved in DMSO (0.1% v/v).

**#**	Test item	HepaRG μg/ml	PHH μg/ml
1	Omeprazole	40	40
2	Carbamazepine	40	40
3	Phenytoin sodium	30	40
4	Penicillin G sodium	40	40
5	Indole carbinol	insoluble (excluded)	
6	Rifabutin	cytotoxic	20
7	Sulfinpyrazone	40	40
8	Bosentan hydrate	40	10
9	Artemisinin	40	40
10	Efavirenz	cytotoxic	2.5
11	Rifampicin	40	40
12	Metoprolol	40	40
13	Sotalol hydrochloride	40	40

### 3.2 Chemical space analysis

**Fig. 4:**
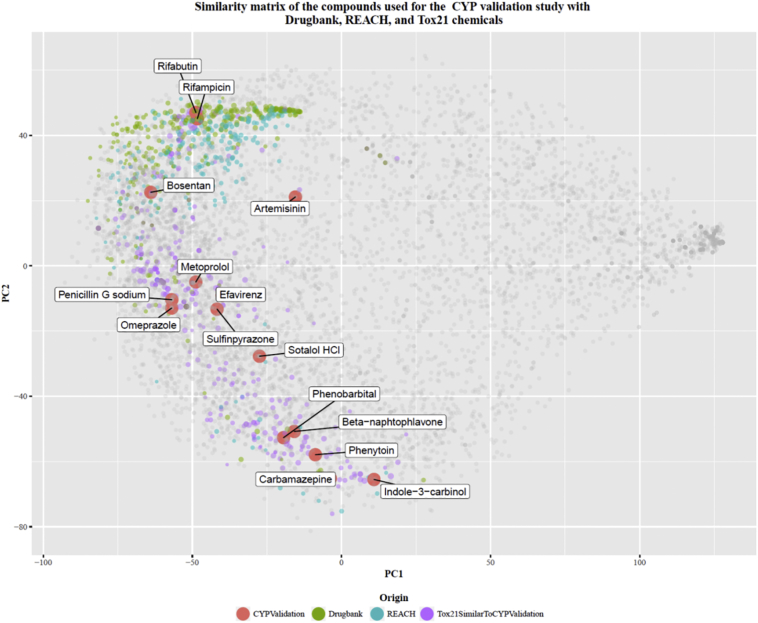
Chemical space of the 15 test/reference items used in the validation study. The axis and positions of the chemicals correspond to the first two principal components of the similarity matrix of the chemicals built using the RDKit (Landrum G. RDKit: Open-source informatics. 2015. http://www.rdkit.org) atomic pairs fingerprints. The chemicals are colour-coded by the list of origin. The sizes of the dots are proportional to their structural similarity to the most similar chemical of the CYP validation study. Chemicals depicted in “grey,” regardless of the list to which they belong, correspond to chemicals with a Tanimoto similarity <0.5 with respect to the validation study chemicals.

The chemicals used in the CYP induction validation study are drugs. However, these are not the only type of chemical that may interact with CYP receptors, as it is not the chemical use that gives them the “ability” to interact with CYP receptors but their chemical structure. The chemical space covered by the 13 test chemicals and the 3 reference chemicals (with rifampicin being both a blind coded (test) chemical and a reference chemical) used in the validation study (Fig. 4) is represented by showing the position of these in the similarity space formed by other chemicals found in relevant lists (i.e. REACH, Drugbank, and Tox21). Once duplicates and chemicals without a defined structure were filtered out, the total number of chemicals conforming the similarity space was 7461.

The chemical space in Fig. 4 shows the chemicals of the lists mentioned above positioned with respect to their structural similarity. The axes of the plot correspond to the first 2 principal components of the similarity matrix calculated using the atomic pairs fingerprints used to describe the chemicals (Landrum G. RDKit: Open-source informatics. 2015. http://www.rdkit.org). In Fig. 4, structurally similar chemicals are placed next to each other and chemicals that are increasingly different are placed further from each other. Indole-3-carbinol and rifabutin, for instance, are placed at the top and bottom of the chemical space. The rest of the validation set chemicals are distributed between these two. This indicates that the structural diversity of the validation set chemicals is high.

Chemicals in Fig. 4 have been coloured by their list of origin. In order to facilitate the visualisation, chemicals that were not similar to any of the CYP induction validation study chemicals, i.e. Tanimoto similarity <0.5, were plotted in grey, regardless of the chemical list to which they belonged.

### 3.3 *In vitro* method performance

#### 3.3.1 Evaluation of CYP induction potential

Based on the solubility and cytotoxicity acceptance criteria, 10 chemicals were further tested with HepaRG cells and 12 with PHH (Table 4). A validation set chemical was classified as an *in vitro* inducer if the CYP induction was ≥2 fold at two or more consecutive concentrations tested. The assigned classifications are reported in Tables 5a for HepaRG, and in Tables 5b for PHH.

**Table 5a:** HepaRG. Assigned classifications (1=Positive=inducer, 0=Negative=non-inducer). The batch is identified by two digits reported above.

	CYP1A2									CYP2B6									CYP3A4								
	Lab 1			Lab 2			Lab 3			Lab 1			Lab 2			Lab 3			Lab 1			Lab 2			Lab 3		
Batch	20	35	36	20	35	36	20	35	36	20	35	36	20	35	36	20	35	36	20	35	36	20	35	36	20	35	36

Omeprazole	1	1	1	1	1	1	1	1	1	0	0	0	0	0	1	1	0	0	0	0	0	0	0	0	0	0	0
Carbamazepine	1	1	1	1	1	1	0	1	1	1	1	1	1	1	1	1	1	1	1	1	0	1	1	1	1	1	1
Phenytoin sodium	1	1	1	1	1	1	1	0	1	1	1	1	1	1	1	1	1	1	1	1	1	1	1	1	1	1	1
Penicillin G sodium	0	0	0	0	0	0	0	0	0	0	0	0	0	0	0	0	0	0	0	0	0	0	0	0	0	0	0
Sulfinpyrazone	1	1	1	1	1	1	1	0	1	0	1	0	1	1	0	0	0	1	1	1	1	1	1	1	1	1	1
Bosentan hydrate	1	1	1	1	1	1	1	1	0	1	1	0	1	0	0	0	1	0	1	1	1	1	1	1	1	1	1
Artemisinin	0	0	0	0	0	0	0	0	0	1	1	1	1	1	1	1	1	1	0	0	0	0	0	0	0	0	0
Rifampicin	1	1	1	1	1	1	1	1	1	0	1	0	1	0	1	0	0	1	1	1	1	1	1	1	1	1	1
Metoprolol	0	0	0	0	1	0	0	0	0	0	0	0	0	0	0	0	0	0	0	0	0	0	0	0	0	0	0
Sotalol hydrochloride	0	0	0	0	0	0	1	0	0	0	0	0	0	0	0	0	0	0	0	0	0	0	0	0	0	0	0

**Table 5b:** PHH. Assigned classifications (1=Positive=inducer, 0=Negative= non-inducer). The batch is identified by three digits reported above.

	CYP1A2									CYP2B6									CYP3A4								
	Lab 1			Lab 2			Lab 3			Lab 1			Lab 2			Lab 3			Lab 1			Lab 2			Lab 3		
Batch	8 0 8	4 0 8	0 6 A	8 0 8	4 0 8	0 6 A	8 0 8	4 0 8	0 6 A	8 0 8	4 0 8	0 6 A	8 0 8	4 0 8	0 6 A	8 0 8	4 0 8	0 6 A	8 0 8	4 0 8	0 6 A	8 0 8	4 0 8	0 6 A	8 0 8	4 0 8	0 6 A

Omeprazole	1	1	1	1	1	1	1	1	1	0	1	1	0	0	1	1	1	1	1	0	1	0	0	1	1	0	1
Carbamazepine	0	1	0	0	0	0	0	0	1	0	1	1	0	1	1	0	1	1	1	1	1	1	1	1	1	1	1
Phenytoin sodium	0	0	1	0	0	1	0	1	1	0	1	1	0	1	1	1	1	1	1	0	1	1	1	1	1	1	1
Penicillin G sodium	0	0	0	0	0	0	0	0	0	0	0	0	0	0	0	0	0	0	0	0	0	0	0	0	0	0	0
Rifabutin	0	1	1	1	0	0	0	0	1	1	0	1	1	0	0	1	1	1	1	0	1	1	1	1	1	1	1
Sulfinpyrazone	0	0	0	0	0	0	0	0	1	1	1	1	1	0	1	1	1	1	1	1	1	1	1	1	1	1	1
Bosentan hydrate	0	0	0	1	0	0	0	0	1	1	1	1	1	0	1	1	1	1	1	1	1	1	1	1	1	1	1
Artemisinin	0	0	0	0	0	0	1	1	0	1	0	0	0	1	1	0	1	1	1	0	1	1	1	1	1	0	1
Efavirenz	0	0	0	0	0	0	0	0	0	1	1	1	0	1	1	0	0	1	1	1	1	1	1	1	1	1	1
Rifampicin	0	0	0	1	0	0	0	0	1	1	1	1	1	0	1	1	1	1	1	1	1	1	1	1	1	1	1
Metoprolol	1	1	0	0	0	0	0	0	1	0	0	0	0	0	0	0	0	1	0	0	0	0	0	1	0	0	1
Sotalol hydrochloride	0	0	0	0	0	0	0	0	0	0	0	0	0	0	0	0	0	1	0	0	0	0	0	0	0	0	0

#### 3.3.2 Reproducibility

Results for between batch reproducibility BBR_L_ and between laboratory reproducibility BLR_B_ are summarised in Table 6a, Table 6b, Table 6c, Table 6d.

**Table 6a:** HepaRG BBR. Proportion of test items with the same classification across three batches for each laboratory.

		CYP1A2			CYP2B6			CYP3A4		
		Lab 1	Lab 2	Lab 3	Lab 1	Lab 2	Lab 3	Lab 1	Lab 2	Lab 3
BBR [%]	per lab	**100%**	**90%**	**50%**	**70%**	**60%**	**60%**	**90%**	**100%**	**100%**
		10/10	9/10	5/10	7/10	6/10	6/10	9/10	10/10	10/10
	overall	**80%**			**63%**			**97%**

**Table 6b:** HepaRG BLR. Proportion of test items with the same classification across three laboratories for each batch.

		CYP1A2			CYP2B6			CYP3A4		
		Batch 1	Batch 2	Batch 3	Batch 1	Batch 2	Batch 3	Batch 1	Batch 2	Batch 3
BLR [%]	per batch	**80%**	**70%**	**90%**	**60%**	**70%**	**70%**	**100%**	**100%**	**90%**
		8/10	7/10	9/10	6/10	7/10	7/10	10/10	10/10	9/10
	overall	**80%**			**67%**			**97%**

**Table 6c:** PHH BBR. Proportion of test items with the same classification across three batches for each laboratory.

		CYP1A2			CYP2B6			CYP3A4		
		Lab 1	Lab 2	Lab 3	Lab 1	Lab 2	Lab 3	Lab 1	Lab 2	Lab 3
BBR [%]	per lab	**67%**	**67%**	**33%**	**58%**	**25%**	**58%**	**67%**	**83%**	**75%**
		8/12	8/12	4/12	7/12	3/12	7/12	8/12	10/12	9/12
	overall	**56%**			**47%**			**75%**

**Table 6d:** PHH BLR. Proportion of test items with the same classification across three laboratories for each batch.

		CYP1A2			CYP2B6			CYP3A4		
		Batch 1	Batch 2	Batch 3	Batch 1	Batch 2	Batch 3	Batch 1	Batch 2	Batch 3
BLR [%]	Per batch	**58%**	**58%**	**50%**	**67%**	**42%**	**67%**	**92%**	**75%**	**92%**
		7/12	7/12	6/12	8/12	5/12	8/12	11/12	9/12	11/12
	Overall	**56%**			**58%**			**86%**		

For all three CYP enzymes, a consistently higher reproducibility for both BBR_L_ and BLR_B_ was obtained for HepaRG cells compared to PHH. This is likely due to the single donor source of the HepaRG cell batches, while PHH originated from three different donors.

For HepaRG cells BBR_L_ values are similar for a given batch except in the case of CYP1A2. The BBR_L_ for CYP3A4 is 90-100%, for CYP2B6 60-70%, and for CYP1A2 between 50% and 100% (Table 6a).

For PHH, the BBR_L_ for CYP3A4 varies from 67% to 83%, for CYP2B6 from 25-58%, and for CYP1A2 between 33% and 67% (Table 6c).

For both HepaRG cells and PHH, the BLR_B_ measures are very similar for a given CYP. For HepaRG cells (Table 6b), the highest values were obtained for CYP3A4 (90-100%), followed by CYP1A2 (70-90%) and finally CYP2B6 (60-70%). For PHH (Table 6d), the highest values were obtained for CYP3A4 (75-92%), followed by CYP1A2 (50-58%) and CYP2B6 (42-67%).

The lower BLR for CYP1A2 in the case of PHH may reflect its higher variation in expression across individuals coupled with the 2-fold threshold definition for induction. The use of a higher cutoff value for induction (e.g. 5-fold) would decrease sensitivity to background noise and probably increase reproducibility for this enzyme.

#### 3.3.3 Predictive capacity

An overview of the predicted and reference classifications for CYP1A2, CYP2B6 and CYP3A4 in PHH and HepaRG cells is presented in Table 7, Table 8, Table 9, Table 10, Table 11, Table 12.

**Table 7:** Prediction of CYP1A2 *in vivo* induction classification (inducer/non-inducer) by HepaRG cells.

#		Induction *in vitro*	F2 (μM)	C_max_ (μM)	C_max_/F2	Prediction *in vivo* inducer	*Induction in vivo*
1	Omeprazole	Yes	12.9	0.68-3.5	0.05-0.3	No	No
2	Carbamazepine	Yes	56.4	39	0.7	Yes	Yes
3	Phenytoin	Yes	36.5	40-80	1.1-2.2	Yes	Yes
4	Penicillin G	No	n.v.	36	n.v.	No	No
7	Sulfinpyrazone	Yes	11	45	4	Yes	Yes
8	Bosentan	Yes	2.60-7.80	5.8	0.45-0.74	Yes	Unknown
9	Artemisinin	No	n.v.	1.0-2.0	n.v.	No	Unknown
11	Rifampicin	Yes	0.20-0.60	8.0-12.0	13-60	Yes	Yes
12	Metoprolol	No	n.v.	0.14-0.38	n.v.	No	No
13	Sotalol	No	n.v.	2	n.v.	No	No

**Table 8:** Prediction of CYP1A2 *in vivo* induction classification (inducer/non-inducer) by PHH.

#		Induction *in vitro*	F2 (μM)	C_max_ (μM)	C_max_/F2	Prediction *in vivo* inducer	Induction *in vivo*
1	Omeprazole	Yes	38.6-118	0.68-3.5	0.005-0.09	No	No
2	Carbamazepine	No	n.v.	39	n.v.	No	Yes
3	Phenytoin	Yes	16.2-48.6	40-80	0.8-4.9	Yes	Yes
4	Penicillin G	No	n.v.	36	n.v.	No	No
6	Rifabutin	Yes	2.2-6.7	0.44	0.06-0.2	No	No
7	Sulfinpyrazone	No	n.v.	45	n.v.	No	Yes
8	Bosentan	No	n.v.	5.8	n.v.	No	Unknown
9	Artemisinin	No	n.v.	1.0-2.0	n.v.	No	Unknown
10	Efavirenz	No	n.v.	9.1-12.6	n.v.	No	Unknown
11	Rifampicin	No	n.v.	8.0-12.0	n.v.	No	Yes
12	Metoprolol	No	n.v.	0.14-0.38	n.v.	No	No
13	Sotalol	No	n.v.	2	n.v.	No	No

**Table 9:** Prediction of CYP2B6 *in vivo* induction classification (inducer/non-inducer) by HepaRG cells.

#		Induction *in vitro*	F2 (μM)	C_max_ (μM)	C_max_/F2	Prediction *in vivo* inducer	Induction *in vivo*
1	Omeprazole	No	n.v.	0.68-3.5	n.v.	No	Unknown
2	Carbamazepine	Yes	18.8	39	2.1	Yes	Yes
3	Phenytoin	Yes	1.35	40-80	29.6-59	Yes	Yes
4	Penicillin G	No	n.v.	36	n.v.	No	No
7	Sulfinpyrazone	Yes	11.0-33.0	45	1.3-4.1	Yes	Unknown
8	Bosentan	Yes	2.60-7.80	5.8	0.7-2.2	Yes	Unknown
9	Artemisinin	Yes	0.58	1.0-2.0	1.7-3.4	Yes	Yes
11	Rifampicin	Yes	0.6-1.80	8.0-12.0	4.4-20	Yes	Yes
12	Metoprolol	No	n.v.	0.14-0.38	n.v.	No	No
13	Sotalol	No	n.v.	2	n.v.	No	No

**Table 10:** Prediction of CYP2B6 *in vivo* induction classification (inducer/non-inducer) by PHH.

#		Induction *in vitro*	F2 (μM)	C_max_ (μM)	C_max_/F2	Prediction *in vivo* inducer	Induction *in vivo*
1	Omeprazole	Yes	38.6-116	0.68-3.5	0.0006-0.09	No	Unknown
2	Carbamazepine	Yes	2.09-6.27	39	66.2-18.7	Yes	Yes
3	Phenytoin	Yes	0.60-48.6	40-80	0.8-133	Yes	Yes
4	Penicillin G	No	n.v.	36	n.v.	No	No
6	Rifabutin	Yes	0.29-0.87	0.44	1.5-2.0	Yes	Unknown
7	Sulfinpyrazone	Yes	33	45	1.3	Yes	Unknown
8	Bosentan	Yes	5.85	5.8	1	Yes	Unknown
9	Artemisinin	Yes	0.58-1.75	1.0-2.0	0.4-3.4	Yes	Yes
10	Efavirenz	Yes	4.69	9.1-12.6	1.9-2.7	Yes	Yes
11	Rifampicin	Yes	0.2	8.0-12.0	40-60	Yes	Yes
12	Metoprolol	No	n.v.	0.14-0.38	n.v.	No	No
13	Sotalol	No	n.v.	2	n.v.	No	No

**Table 11:** Prediction of CYP3A4 *in vivo* induction classification (inducer/non-inducer) by HepaRG cells.

#		Induction *in vitro*	F2 (μM)	C_max_ (μM)	C_max_/F2	Prediction *in vivo* inducer	Induction *in vivo*
1	Omeprazole	No	n.v.	0.68-3.5	n.v.	No	No
2	Carbamazepine	Yes	18.8	39	2	Yes	Yes
3	Phenytoin	Yes	12.2	40-80	3.2-6.5	Yes	Yes
4	Penicillin G	No	n.v.	36	n.v.	No	No
7	Sulfinpyrazone	Yes	11	45	4.1	Yes	Yes
8	Bosentan	Yes	0.29	5.8	20	Yes	Yes
9	Artemisinin	No	n.v.	1.0-2.0	n.v.	No	Yes/No
11	Rifampicin	Yes	0.2-0.6	8.0-12.0	40-13	Yes	Yes
12	Metoprolol	No	n.v.	0.14-0.38	n.v.	No	No
13	Sotalol	No	n.v.	2	n.v.	No	No

**Table 12:** Prediction of CYP3A4 *in vivo* induction classification (inducer/non-inducer) by PHH.

#		Induction *in vitro*	F2 (μM)	C_max_ (μM)	C_max_/F2	Prediction *in vivo* inducer	Induction *in vivo*
1	Omeprazole	Yes	38.7-117	0.68-3.5	0.017-0.09	No	No
2	Carbamazepine	Yes	6.27-18.	39	6.2-2.2	Yes	Yes
3	Phenytoin	Yes	1.80-16.2	40-80	2.4-44	Yes	Yes
4	Penicillin G	No	112	36	0.32	No	No
6	Rifabutin	Yes	0.1	0.44	4.4	Yes	Yes
7	Sulfinpyrazone	Yes	3.66	45	12.2	Yes	Yes
8	Bosentan	Yes	0.65-1.95	5.8	2.9-8.9	Yes	Yes
9	Artemisinin	Yes	5.25-47.2	1.0-2.0	0.02-0.38	No	Yes/No
10	Efavirenz	Yes	14.1	9.1-12.6	0.65-0.89	Yes	Yes
11	Rifampicin	Yes	0.2	8.0-12.0	40-60	Yes	Yes
12	Metoprolol	No	n.v.	0.14-0.38	n.v.	No	No
13	Sotalol	No	n.v.	2	n.v.	No	No

Omeprazole is not a CYP1A2 inducer *in vivo* at normal doses (20-40 mg) while it has been found to be a weak inducer at 120 mg doses or in poor metabolizers (40 mg dose) reaching high plasma levels (Andersson, 1996). The Cmax values used in Table 7, Table 8, Table 9, Table 10, Table 11, Table 12 refer to a normal dose. *In vitro* omeprazole has been used in other studies as a positive control at concentrations not relevant for a normal dose.

HepaRG cells predicted human *in vivo* CYP1A2 induction for the four true positives (Table 7). Four *in vivo* negatives were also correctly classified. For bosentan and artemisinin, *in vivo* data were lacking. For bosentan CYP1A2 induction would be expected from the Cmax/F2 ratio (0.45-0.74) >0.5, but artemisinin would not be expected to induce CYP1A2 *in vivo*.

PHH correctly predicted CYP1A2 induction for one of the four *in vivo* inducers: phenytoin (Table 8). All five of the *in vivo* negatives were correctly predicted.

For artemisinin, PHH predicted no CYP1A2 induction. However, the correctness of the prediction cannot be evaluated due to the *in vitro* variability and absence of human *in vivo* data. Clinical studies were also lacking for bosentan and efavirenz (both indicated as non-inducers by PHH) precluding verification of *in vitro* predictive capacity for CYP1A2 induction. PHH misclassified rifampicin as a non-inducer of CYP1A2, contrary to published *in vivo* data (Köhle and Bock, 2009; Hoffmann et al., 2014; Kanebratt and Andersson, 2008; Derungs et al., 2016). However, rifampicin induction of CYP1A *in vivo* is weak and difficult to capture in PHH (Moscovitz et al., 2018; Rae et al., 2001). The discrepancy may be also related to apparent variability of individual hepatocyte batches (Abadie-Viollon et al., 2010; Yajima et al., 2014). PHH misclassified also sulfinpyrazone and carbamazepine as a non-inducer of CYP1A2, contrary to published *in vivo* data.

For CYP2B6, the four inducers carbamazepine, phenytoin, artemisinin, and rifampicin were correctly classified by HepaRG cells; the three *in vivo* negatives (penicillin, metoprolol and sotalol) were also correctly predicted (Table 9).

In the absence of human *in vivo* data on CYP2B6 induction for sulfinpyrazone and bosentan, both were predicted as positive inducers by HepaRG cells at clinically relevant doses. In HepaRG cells omeprazole was predicted as a non-inducer of human CYP2B6 consistent with observations *in vivo* at clinically relevant doses. CYP2B6 induction by sulfinpyrazone has been demonstrated in human hepatocytes (Faucette et al., 2004) supporting the positive prediction by HepaRG cells. Assuming validity of the *in vitro* results for omeprazole and sulfinpyrazone, the positive outcome for bosentan may similarly be inferred as true.

The results for CYP2B6 induction by PHH are the same as for HepaRG. Rifabutin (only tested in PHH) also induced CYP2B6, although the absence of *in vivo* human data precluded direct comparison (Table 10). The positive predictions for carbamazepine, phenytoin, artemisinin, efavirenz and rifampicin, and the negative results for penicillin, metoprolol and sotalol, are concordant with their respective positive/negative classifications *in vivo*.

Predictions of CYP3A4 induction were correct for both HepaRG cells and PHH, except for artemisinin (Table 11, Table 12). The four non-inducers were also correctly predicted by both test systems.

Although artemisinin was indicated as negative *in vitro* inducer by HepaRG cells and as positive *in vitro* inducer by PHH, the latter was observed at concentrations above Cmax for human *in vivo*. On this basis, artemisinin was predicted to be a non-inducer. Variability is also evident in clinical studies on CYP3A4 induction by artemisinin: a study of midazolam metabolite/parent ratio indicated CYP3A4 induction (Asimus et al., 2007), whereas no CYP3A4 induction by the omeprazole sulfone formation and cortisol metabolic ratio was reported (Svensson et al., 1998).

## 4 Discussion

CYP induction, requiring *de novo* protein synthesis, is a sensitive biomarker for phenotypic hepatic metabolic competence. For the first time, PHH and HepaRG cells have been formally validated as metabolically competent test systems for the functional assessment of CYP1A2, CYP2B6 and CYP3A4 induction. The measurement of functional CYP enzyme induction (i.e. catalytic activity) is considered more informative than measurements of mRNA, since correlations between the CYP-selective activity and the specific CYP mRNA level are frequently poor or lacking (Abass et al., 2012; Choi et al., 2013; Mwinyi et al., 2011; Nakajima and Yokoi, 2011; Surapureddi et al., 2011).

The ring trial results show adequate (Table 6a, Table 6b, Table 6c, Table 6d) within and between laboratory reproducibility, demonstrating that both methods are transferable to laboratories experienced in cell culture techniques and analytical chemistry. The design and conduct of the validation study followed best practices. For example, the methods avoided the use of serum, which has a complex composition and introduces undefined components into the medium, thereby affecting reproducibility of results. Provisions such as this are now explicitly documented in the recently published OECD guidance on Good In Vitro Method Practices (GIVIMP; OECD, 2018).

The results also show that the two *in vitro* methods provide reasonable predictions of the *in vivo* CYP enzyme induction of chemicals (Table 6a, Table 6b, Table 6c, Table 6d), allowing the choice of test system to depend upon the assessment context (discussed further below). Both test systems correctly responded to the reference inducers (BNF, PB, and RIF) and correctly predicted *in vivo* human CYP induction for all the blind coded chemicals tested, except for carbamazepine, sulfinpyrazone and rifampicin in PHH (Table 13). In some cases, the absence of adequate human data (i.e. the available *in vivo* data for CYP3A4 induction by artemisinin were inconsistent) precluded an assessment of predictivity (yellow boxes in Table 13).

**Table 13:**
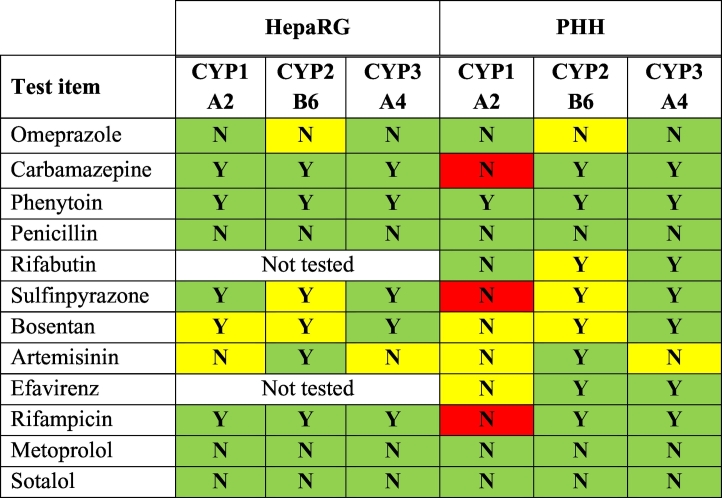
Summary predictive capacity of HepaRG cells and PHH for CYP1A2, CYP2B6 and CYP3A4 induction by test chemicals.

Colour shading key: green: correct prediction (true positive or true negative); yellow: unconfirmed (no or unreliable or inconsistent *in vivo* data) or ambiguous; red: incorrect prediction.

Although the validation set of chemicals was of limited size due to the availability of human data, the chemical space analysis shows that these chemicals span a relative large area of the chemical space formed by REACH registered substances, Drugbank approved drugs and some Tox21 chemicals. This suggests that that the CYP induction methods may be applicable to a structurally diverse range of chemicals.

The mechanistic relevance (metabolic competence) of the *in vitro* methods is based on the fact that the entire catalytic machinery (transporters, nuclear receptors, transcription and translation into a functional enzyme) is present and functional in the test systems.

In general, the functional measurement of CYP induction should be sufficient. However, the parallel measurement of mRNA might be warranted in some specific cases, for example when the chemical is both a CYP inhibitor and inducer (Einolf et al., 2014). Xing et al. (2012) describes the auto-induction phenomenon for artemisinin following observation of more induction of CYP3A4 transcripts than activity as the artemisinin concentration increased. This supports the hypothesis that at higher artemisinin concentrations weak or slow inactivation may dampen the increase in CYP3A4 activity relative to mRNA transcripts.

CYP induction is a concentration-dependent process. Therefore, the assessment of the predictive capacity of human *in vitro* methods needs to take into account realistic human *in vivo* concentrations of a chemical. This should preferably be the concentration at the site of action, but plasma concentration generally serves as a more convenient and suitable exposure metric. A case in point was omeprazole, indicated by PHH and HepaRG cells as an *in vitro* inducer of all three CYP isoforms. However, the concentrations producing 2-fold induction significantly exceeded the clinical Cmax, and consequently omeprazole was concluded as non-inducer. Omeprazole has been demonstrated to induce CYP1A2 in humans, measured by caffeine metabolism or phenacetin clearance, but only at elevated doses above the clinical norm, or in subjects with poor omeprazole metabolism (Rost et al., 1994 and Rost et al., 1999). Nevertheless, the omeprazole example illustrates the need for rational comparison. With chemicals other than drugs, concentrations could be obtained from human biomonitoring studies or, if an actual measured concentration is not available, from a calculation based on external exposure assumptions, for example by using physiological biokinetic (e.g. PBPK) models (Bessems et al., 2014).

For the three validation set chemicals acting as negative controls *in vivo*, i.e. penicillin, metoprolol, and sotalol, *in vitro* results were concordant, although metoprolol demonstrated some response in isolated cases with PHH. Despite the fact concentration-response curves were consistent in these isolated cases, concentrations for induction were much higher than clinical Cmax concentrations. Therefore, metoprolol was predicted to be a non-inducer *in vivo*. PHH missed the *in vivo* prediction for CYP1A2 induction by carbamazepine, sulfinpyrazone and rifampicin, possibly related to variability of individual hepatocyte batches.

The choice of PHH or HepaRG cells as test system is largely dependent on the application. PHH have long been limited by availability. With the current successes in cryopreserving (Abadie-Viollon et al., 2010; Alexandre et al., 2012) progress has been made allowing quality control and generation of test system characterisation data by commercial providers. Inevitably, PHH are subject to variability among individual donors (Costa et al., 2014) which might be desirable for certain applications where population variability data are necessary. For other routine chemical testing applications, immortalised cell lines have been proposed. Among these, HepaRG cells provide an immortalised hepatocyte human cell line with relevant *in vivo* functions (Aninat et al., 2006; Le Vee et al., 2006) and continuity of batch consistency. The biotransformation enzyme composition of HepaRG cells can be sustained over weeks (Guillouzo et al., 2007).

Human derived metabolically competent test systems are of particular relevance for human safety assessment since there are well described species differences in Phase I enzyme induction and metabolism (Martignoni et al., 2006, Kedderis and Lipscomb, 2001), metabolic stability and metabolite identification (Pelkonen et al., 2009), and in CAR, PXR and AhR receptor activation (Kretschmer and Baldwin, 2005; Kiyosawa et al., 2008; Köhle and Bock, 2009; Abass et al., 2012 and Fujiwara et al., 2012).

## 5 Conclusions and recommendations

The present validation study shows that cryopreserved PHH and cryopreserved HepaRG cells are reliable and relevant *in vitro* methods for the assessment of human CYP enzyme induction. These methods may play a role in regulatory risk assessment by contributing information on metabolism, thyroid disruption, or as indicators of nuclear-receptor mediated dysregulation of biochemical pathways. Assessing the toxicological relevance of the two methods, and in particular the more standardised HepaRG cell method, in specific regulatory assessment contexts was not within the scope of the present validation study. This should, however, be the focus of further investigations.

**Fig. 5:**
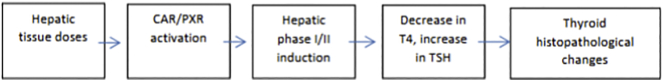
Example of a postulated mode of action for increase in thyroid hormone metabolism (ECHA and EFSA, 2018)

CYP induction is a nuclear receptor-mediated process and following AhR, PXR and CAR activation, xenobiotics may dysregulate an array of fundamental cell functions (Sueyoshi et al., 2014; Dingemans et al., 2016; Ovchinnikov et al., 2018; Hakkola et al., 2018; Sanders et al., 2005). CYP induction may therefore serve as a biomarker of nuclear receptor activation. The induction of Phase I enzymes in the liver is considered a potential key event in endocrine (thyroid) disruption in the recently published ECHA/EFSA Guidance (ECHA and EFSA, 2018). In particular, when there is evidence that these receptors are involved in pathways for which specific measurement methods are lacking (e.g. induction of Phase II enzymes for glucuronidation and sulfation; Kodama and Negishi, 2013), the two validated human CYP induction in vitro methods could be used as surrogates (EFSA, 2019). To study thyroid hormone metabolism following chemical exposure, a battery of validated in vitro methods is needed to investigate the effect of inhibition and induction of Phase I and Phase II biotransformation enzymes and the clearance levels of thyroid hormones (OECD, 2014; OECD, 2017 and Fig. 5)

Following the analysis of data generated and additional peer reviewed evidence, the following recommendations are proposed for the practical conduct of CYP enzyme induction studies: a) CYP induction can be measured at a phenotypic level (i.e. enzyme activity), b) CYP enzyme induction should be measured in human derived metabolically competent test systems; c) cryopreserved HepaRG cells are comparable to cryopreserved PHH in predicting CYP enzyme induction, representing a substitute/complementary *in vitro* system for CYP induction studies; d) 2-fold induction is an acceptable threshold for positive identification of *in vitro* CYP inducers; and e) to reduce the risk of false positives, a concentration-dependent response (i.e. at least two consecutive concentrations generating 2-fold induction response) should be observed to classify a compound as an *in vitro* inducer.

## Disclaimer

The views expressed in this article are those of the authors and do not necessarily represent the views or policies of the European Commission.

## Footnotes

1 PXR, CAR and AhR are often called ‘nuclear’ receptors, although AhR belongs actually to the family of basic-helix/loop/helix (bHLH)-receptors (bHLHe76) and only PXR and CAR belong to the family of nuclear receptors (NR1I2 for PXR and NR1I3 for CAR).

2 HepaRG: GlutaMAX™ with serum-free supplement PHH: HMM (hepatocyte maintenance medium)

3 probe substrates are not 100% selective for the assigned CYP enzyme, but each CYP enzyme is the primary and major catalyst of the probe reaction.
